# Bis(5-bromo­pyridine-2-carboxyl­ato-κ*O*)triphenyl­anti­mony(V)

**DOI:** 10.1107/S1600536808033783

**Published:** 2008-11-08

**Authors:** Li Quan, Handong Yin, Daqi Wang

**Affiliations:** a College of Chemistry and Chemical Engineering, Liaocheng University, Shandong 252059, People’s Republic of China

## Abstract

In the title compound, [Sb(C_6_H_5_)_3_(C_6_H_3_BrNO_2_)_2_], the Sb center has a distorted trigonal–bipyramidal geometry, with two carboxyl­ate O atoms of two 5-bromo­pyridine-2-carboxyl­ate ligands in equatorial positions and three phenyl ligands in axial positions. The crystal structure is stabilized by C—H⋯Br hydrogen bonds and inter­molecular C—Br⋯π inter­actions [C⋯π = 3.57 (1) Å].

## Related literature

For the synthesis and structures of related triphenyl­anti­mony compounds, see: Yin *et al.* (2008 ▶); Chaudhari *et al.* (2007 ▶); Mahon *et al.* (1998 ▶); Quan *et al.* (2008 ▶).
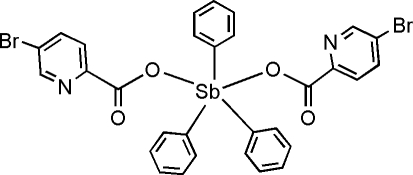


## Experimental

### 

#### Crystal data

[Sb(C_6_H_5_)_3_(C_6_H_3_BrNO_2_)_2_]
*M* *_r_* = 755.06
Orthorhombic, 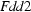

*a* = 20.597 (2) Å
*b* = 13.057 (1) Å
*c* = 20.541 (2) Å
*V* = 5524.2 (9) Å^3^
*Z* = 8
Mo *K*α radiation
μ = 3.93 mm^−1^
*T* = 298 (2) K
0.43 × 0.37 × 0.20 mm

#### Data collection

Siemens SMART diffractometer
Absorption correction: multi-scan (*SADABS*; Sheldrick, 1996 ▶) *T*_min_ = 0.204, *T*_max_ = 0.460
5869 measured reflections
2564 independent reflections
1861 reflections with *I* > 2σ(*I*)
*R*_int_ = 0.081

#### Refinement

*R*[*F* ^2^ > 2σ(*F* ^2^)] = 0.060
*wR*(*F* ^2^) = 0.166
*S* = 1.01
2564 reflections
178 parameters
1 restraint
H-atom parameters constrained
Δρ_max_ = 1.02 e Å^−3^
Δρ_min_ = −0.86 e Å^−3^
Absolute structure: Flack (1983 ▶), 1172 Friedel pairs
Flack parameter: 0.02 (3)

### 

Data collection: *SMART* (Siemens, 1996 ▶); cell refinement: *SAINT* (Siemens, 1996 ▶); data reduction: *SAINT*; program(s) used to solve structure: *SHELXS97* (Sheldrick, 2008 ▶); program(s) used to refine structure: *SHELXL97* (Sheldrick, 2008 ▶); molecular graphics: *ORTEP-3* (Farrugia, 1997 ▶) and *DIAMOND* (Brandenburg, 1998 ▶); software used to prepare material for publication: *SHELXTL* (Sheldrick, 2008 ▶).

## Supplementary Material

Crystal structure: contains datablocks I, global. DOI: 10.1107/S1600536808033783/lx2073sup1.cif

Structure factors: contains datablocks I. DOI: 10.1107/S1600536808033783/lx2073Isup2.hkl

Additional supplementary materials:  crystallographic information; 3D view; checkCIF report

## Figures and Tables

**Table 1:** Hydrogen-bond geometry (Å, °)

*D*—H⋯*A*	*D*—H	H⋯*A*	*D*⋯*A*	*D*—H⋯*A*
C10—H10⋯Br^i^	0.93	2.90	3.69 (2)	144

Symmetry code: (i) 
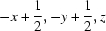
.

**Fig. 1.**
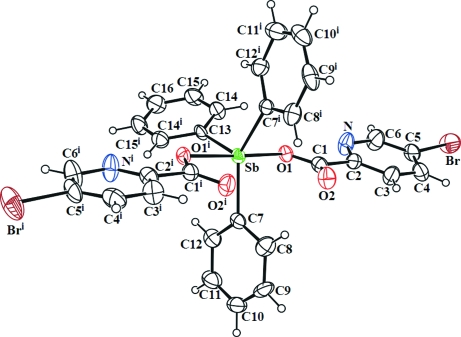
The molecular structure of the title compound, showing displacement ellipsoids drawn at the 30% probability level. [Symmetry code: (i) -x+1, -y+1, z.]

**Fig. 2.**
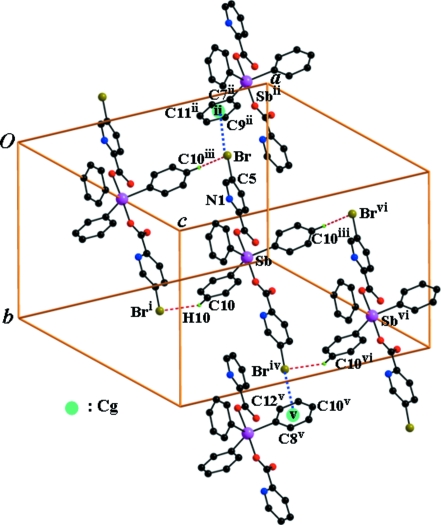
C—H···Br and C—Br···π interactions (dotted lines) in the title compound. Cg denotes the ring centroid. [Symmetry code: (i) -x+1/2, -y+1/2, z; (ii) x, y-1, z; (iii)-x+1/2, -y+1/2, z; (iv) -x+1, -y+1, z; (v) -x+1, -y+2, z; (vi) x+1/2, y+1/2, z.]

## supplementary crystallographic information

### Comment

The triphenylantimony compound containing the heterocyclic pyridine carboxylate skeleton show some potential biological activity (Yin *et al.*,  2008) and we have synthesized the title compound (I) and report its crystal structure here.

As shown in Fig. 1, the Sb atom is five-coordinated by the three phenyl C atoms and the two carboxylate O atoms. The average distance of Sb—C (2.10 Å) in the (I) is shorter than the average distance of S—C (2.225 Å; Mahon *et al.*,  1998). The average distance of Sb—O (2.146 Å) in the (I) is equal to the average distance of Sb—O (2.145 Å; Chaudhari *et al.*, 2007). The crystal structure is stabilized by intermolecular C—H···Br hydrogen bonds (Fig. 2 and Table 1; symmetry code as in Fig. 2). In addition, the crystal structure exhibits C—Br···π interactions, with a C5—Br···Cg^ii^ separation of  3.57 (1) Å (Fig. 2; Cg is the centroid of the C7-C12 benzene ring, symmetry code as in Fig. 2).

### Experimental

5-bromopyridine-2-carboxylic acid (0.061 g, 0.3 mmol) and sodium methoxide (0.6 ml, 0.3 mmol) was added to a stirring solution containing triphenylantimonydichloride (0.064 g, 0.15 mmol) in toluene (25 ml). After refluxing for 8 h, the colorless solution was obtained and then filtered. The solvent was gradually removed by evaporation under vacuum until the white solid is obtained. The solid was recrystallized from petroleum ether/dichoromethane (1:1) to give colorless crystals.

### Refinement

All H atoms were positioned geometrically and refined as riding atoms, with C—H = 0.93 Å, Uiso(H) = 1.2Ueq(C).

### Crystal data

**Table:**

[Sb(C_6_H_5_)_3_(C_6_H_3_BrNO_2_)_2_]	*F*_000_ = 2944
*M**_r_* = 755.06	*D*_x_ = 1.816 Mg m^−^^3^
Orthorhombic, *F**d**d*2	Mo *K*α radiation λ = 0.71073 Å
Hall symbol: f 2 -2d	Cell parameters from 2025 reflections
*a* = 20.597 (2) Å	θ = 2.8–24.1º
*b* = 13.057 (1) Å	µ = 3.93 mm^−^^1^
*c* = 20.541 (2) Å	*T* = 298 (2) K
*V* = 5524.2 (9) Å^3^	Block, colorless
*Z* = 8	0.43 × 0.37 × 0.20 mm

### Data collection

**Table:**

Siemens SMART diffractometer	2564 independent reflections
Radiation source: fine-focus sealed tube	1861 reflections with *I* > 2σ(*I*)
Monochromator: graphite	*R*_int_ = 0.081
Detector resolution: 20.0 pixels mm^-1^	θ_max_ = 26.0º
*T* = 298(2) K	θ_min_ = 2.1º
φ and ω scans	*h* = −23→25
Absorption correction: multi-scan(SADABS; Sheldrick, 1996)	*k* = −16→12
*T*_min_ = 0.204, *T*_max_ = 0.460	*l* = −24→25
5869 measured reflections	

### Refinement

**Table:**

Refinement on *F*^2^	Hydrogen site location: inferred from neighbouring sites
Least-squares matrix: full	H-atom parameters constrained
*R*[*F*^2^ > 2σ(*F*^2^)] = 0.060	*w* = 1/[σ^2^(*F*_o_^2^) + (0.0963*P*)^2^] where *P* = (*F*_o_^2^ + 2*F*_c_^2^)/3
*wR*(*F*^2^) = 0.166	(Δ/σ)_max_ < 0.001
*S* = 1.01	Δρ_max_ = 1.02 e Å^−^^3^
2564 reflections	Δρ_min_ = −0.86 e Å^−^^3^
178 parameters	Extinction correction: none
1 restraint	Absolute structure: Flack (1983), 1172 Friedel pairs
Primary atom site location: structure-invariant direct methods	Flack parameter: 0.02 (3)
Secondary atom site location: difference Fourier map	

### Special details

**Table:**

Geometry. All esds (except the esd in the dihedral angle between two l.s. planes) are estimated using the full covariance matrix. The cell esds are taken into account individually in the estimation of esds in distances, angles and torsion angles; correlations between esds in cell parameters are only used when they are defined by crystal symmetry. An approximate (isotropic) treatment of cell esds is used for estimating esds involving l.s. planes.
Refinement. Refinement of F^2^ against ALL reflections. The weighted R-factor wR and goodness of fit S are based on F^2^, conventional R-factors R are based on F, with F set to zero for negative F^2^. The threshold expression of F^2^ > 2sigma(F^2^) is used only for calculating R-factors(gt) etc. and is not relevant to the choice of reflections for refinement. R-factors based on F^2^ are statistically about twice as large as those based on F, and R- factors based on ALL data will be even larger.

### Fractional atomic coordinates and isotropic or equivalent isotropic displacement parameters (Å^2^)

**Table:**

	*x*	*y*	*z*	*U*_iso_*/*U*_eq_	
Sb	0.5000	0.5000	0.64254 (4)	0.0395 (3)	
Br	0.38657 (9)	−0.14610 (10)	0.69850 (11)	0.1021 (8)	
N	0.4328 (6)	0.1462 (8)	0.6388 (6)	0.074 (3)	
O1	0.4633 (4)	0.3464 (5)	0.6376 (4)	0.0449 (17)	
O2	0.4672 (5)	0.3511 (7)	0.7460 (5)	0.064 (2)	
C1	0.4568 (5)	0.3068 (9)	0.6937 (7)	0.049 (3)	
C2	0.4353 (5)	0.1947 (9)	0.6944 (7)	0.052 (3)	
C3	0.4223 (7)	0.1484 (13)	0.7532 (7)	0.065 (4)	
H3	0.4257	0.1860	0.7916	0.078*	
C4	0.4047 (7)	0.0490 (12)	0.7557 (8)	0.069 (4)	
H4	0.3932	0.0178	0.7947	0.083*	
C5	0.4046 (7)	−0.0025 (8)	0.6998 (8)	0.064 (4)	
C6	0.4174 (9)	0.0468 (10)	0.6421 (10)	0.084 (5)	
H6	0.4153	0.0094	0.6036	0.101*	
C7	0.4109 (5)	0.5538 (8)	0.6790 (6)	0.044 (3)	
C8	0.3959 (7)	0.5504 (11)	0.7467 (7)	0.065 (3)	
H8	0.4256	0.5260	0.7770	0.078*	
C9	0.3346 (8)	0.5853 (12)	0.7657 (9)	0.069 (5)	
H9	0.3235	0.5854	0.8096	0.083*	
C10	0.2904 (7)	0.6196 (10)	0.7200 (10)	0.071 (4)	
H10	0.2495	0.6412	0.7335	0.085*	
C11	0.3054 (7)	0.6225 (11)	0.6559 (9)	0.074 (4)	
H11	0.2753	0.6456	0.6255	0.089*	
C12	0.3667 (6)	0.5902 (9)	0.6362 (7)	0.058 (3)	
H12	0.3776	0.5938	0.5923	0.070*	
C13	0.5000	0.5000	0.5399 (8)	0.043 (4)	
C14	0.5053 (6)	0.4088 (10)	0.5057 (6)	0.058 (3)	
H14	0.5082	0.3467	0.5277	0.070*	
C15	0.5062 (7)	0.4116 (12)	0.4393 (6)	0.068 (4)	
H15	0.5112	0.3508	0.4162	0.082*	
C16	0.5000	0.5000	0.4066 (10)	0.074 (6)	
H16	0.5000	0.5000	0.3613	0.089*	

### Atomic displacement parameters (Å^2^)

**Table:**

	*U*^11^	*U*^22^	*U*^33^	*U*^12^	*U*^13^	*U*^23^
Sb	0.0407 (5)	0.0310 (4)	0.0468 (5)	−0.0008 (5)	0.000	0.000
Br	0.1069 (13)	0.0400 (8)	0.159 (2)	−0.0140 (8)	0.0465 (13)	0.0023 (10)
N	0.118 (10)	0.045 (6)	0.058 (7)	−0.023 (6)	0.010 (8)	−0.004 (6)
O1	0.053 (4)	0.029 (4)	0.052 (5)	−0.004 (3)	−0.001 (4)	−0.002 (4)
O2	0.084 (6)	0.047 (5)	0.060 (5)	−0.011 (5)	−0.010 (5)	−0.005 (4)
C1	0.046 (6)	0.038 (6)	0.064 (8)	−0.001 (5)	−0.004 (6)	−0.004 (6)
C2	0.050 (7)	0.043 (6)	0.064 (8)	0.000 (5)	0.010 (7)	0.005 (6)
C3	0.078 (10)	0.060 (9)	0.057 (9)	−0.007 (7)	0.012 (8)	0.005 (7)
C4	0.070 (9)	0.052 (8)	0.086 (11)	0.009 (7)	0.001 (8)	0.017 (8)
C5	0.069 (9)	0.023 (6)	0.101 (12)	−0.005 (5)	0.014 (8)	0.003 (8)
C6	0.123 (13)	0.041 (7)	0.089 (10)	−0.010 (8)	0.028 (12)	−0.007 (9)
C7	0.039 (6)	0.029 (5)	0.064 (8)	−0.004 (4)	−0.004 (6)	−0.004 (5)
C8	0.075 (9)	0.067 (9)	0.053 (8)	−0.009 (8)	0.005 (7)	−0.009 (7)
C9	0.078 (10)	0.054 (8)	0.075 (10)	−0.010 (8)	0.046 (9)	−0.017 (7)
C10	0.053 (8)	0.049 (8)	0.111 (14)	0.002 (6)	0.026 (9)	−0.005 (8)
C11	0.052 (8)	0.066 (9)	0.105 (14)	0.008 (6)	−0.002 (9)	0.004 (8)
C12	0.056 (7)	0.052 (7)	0.068 (8)	−0.005 (5)	−0.007 (7)	−0.001 (6)
C13	0.052 (9)	0.022 (7)	0.056 (9)	0.009 (7)	0.000	0.000
C14	0.068 (8)	0.050 (7)	0.057 (8)	0.009 (6)	−0.006 (7)	0.003 (6)
C15	0.089 (10)	0.067 (9)	0.049 (7)	0.022 (7)	0.007 (8)	−0.014 (6)
C16	0.085 (14)	0.091 (16)	0.045 (10)	0.019 (12)	0.000	0.000

### Geometric parameters (Å, °)

**Table:**

Sb—C7	2.103 (11)	C7—C8	1.425 (19)
Sb—C7^i^	2.103 (11)	C8—C9	1.40 (2)
Sb—C13	2.108 (17)	C8—H8	0.9300
Sb—O1^i^	2.146 (7)	C9—C10	1.38 (2)
Sb—O1	2.146 (7)	C9—H9	0.9300
Br—C5	1.911 (10)	C10—C11	1.35 (2)
N—C2	1.309 (16)	C10—H10	0.9300
N—C6	1.337 (17)	C11—C12	1.39 (2)
O1—C1	1.270 (16)	C11—H11	0.9300
O2—C1	1.238 (16)	C12—H12	0.9300
C1—C2	1.529 (17)	C13—C14^i^	1.387 (16)
C2—C3	1.377 (19)	C13—C14	1.387 (16)
C3—C4	1.35 (2)	C14—C15	1.364 (19)
C3—H3	0.9300	C14—H14	0.9300
C4—C5	1.33 (2)	C15—C16	1.342 (18)
C4—H4	0.9300	C15—H15	0.9300
C5—C6	1.37 (2)	C16—C15^i^	1.342 (18)
C6—H6	0.9300	C16—H16	0.9300
C7—C12	1.352 (17)		
			
C7—Sb—C7^i^	138.2 (7)	C12—C7—C8	120.0 (12)
C7—Sb—C13	110.9 (3)	C12—C7—Sb	118.2 (10)
C7^i^—Sb—C13	110.9 (3)	C8—C7—Sb	121.8 (10)
C7—Sb—O1^i^	90.7 (3)	C9—C8—C7	117.3 (15)
C7^i^—Sb—O1^i^	91.2 (4)	C9—C8—H8	121.4
C13—Sb—O1^i^	87.3 (2)	C7—C8—H8	121.4
C7—Sb—O1	91.2 (4)	C10—C9—C8	120.7 (14)
C7^i^—Sb—O1	90.7 (3)	C10—C9—H9	119.6
C13—Sb—O1	87.3 (2)	C8—C9—H9	119.6
O1^i^—Sb—O1	174.6 (5)	C11—C10—C9	121.2 (13)
C2—N—C6	115.8 (13)	C11—C10—H10	119.4
C1—O1—Sb	112.0 (7)	C9—C10—H10	119.4
O2—C1—O1	125.4 (11)	C10—C11—C12	118.8 (15)
O2—C1—C2	119.3 (12)	C10—C11—H11	120.6
O1—C1—C2	115.3 (11)	C12—C11—H11	120.6
N—C2—C3	123.1 (12)	C7—C12—C11	121.9 (15)
N—C2—C1	117.8 (11)	C7—C12—H12	119.1
C3—C2—C1	119.0 (13)	C11—C12—H12	119.1
C4—C3—C2	120.5 (14)	C14^i^—C13—C14	119.1 (16)
C4—C3—H3	119.7	C14^i^—C13—Sb	120.4 (8)
C2—C3—H3	119.7	C14—C13—Sb	120.4 (8)
C5—C4—C3	117.0 (14)	C15—C14—C13	119.0 (13)
C5—C4—H4	121.5	C15—C14—H14	120.5
C3—C4—H4	121.5	C13—C14—H14	120.5
C4—C5—C6	120.4 (12)	C16—C15—C14	121.5 (14)
C4—C5—Br	120.5 (12)	C16—C15—H15	119.2
C6—C5—Br	119.0 (11)	C14—C15—H15	119.2
N—C6—C5	123.0 (16)	C15^i^—C16—C15	119.8 (18)
N—C6—H6	118.5	C15^i^—C16—H16	120.1
C5—C6—H6	118.5	C15—C16—H16	120.1
			
C7—Sb—O1—C1	−72.0 (8)	C13—Sb—C7—C8	172.8 (9)
C7^i^—Sb—O1—C1	66.3 (8)	O1^i^—Sb—C7—C8	−99.8 (11)
C13—Sb—O1—C1	177.2 (7)	O1—Sb—C7—C8	85.2 (10)
Sb—O1—C1—O2	3.2 (15)	C12—C7—C8—C9	0.4 (19)
Sb—O1—C1—C2	−176.0 (7)	Sb—C7—C8—C9	−178.2 (10)
C6—N—C2—C3	−1(2)	C7—C8—C9—C10	1(2)
C6—N—C2—C1	176.3 (13)	C8—C9—C10—C11	−1(2)
O2—C1—C2—N	−171.3 (13)	C9—C10—C11—C12	0(2)
O1—C1—C2—N	7.8 (15)	C8—C7—C12—C11	−1.8 (19)
O2—C1—C2—C3	5.8 (17)	Sb—C7—C12—C11	176.8 (10)
O1—C1—C2—C3	−175.0 (12)	C10—C11—C12—C7	2(2)
N—C2—C3—C4	−1(2)	C7—Sb—C13—C14^i^	63.8 (7)
C1—C2—C3—C4	−178.4 (12)	C7^i^—Sb—C13—C14^i^	−116.2 (7)
C2—C3—C4—C5	4(2)	O1^i^—Sb—C13—C14^i^	−25.9 (7)
C3—C4—C5—C6	−5(2)	O1—Sb—C13—C14^i^	154.1 (7)
C3—C4—C5—Br	175.5 (11)	C7—Sb—C13—C14	−116.2 (7)
C2—N—C6—C5	0(2)	C7^i^—Sb—C13—C14	63.8 (7)
C4—C5—C6—N	3(3)	O1^i^—Sb—C13—C14	154.1 (7)
Br—C5—C6—N	−177.5 (13)	O1—Sb—C13—C14	−25.9 (7)
C7^i^—Sb—C7—C12	174.2 (9)	C14^i^—C13—C14—C15	1.0 (10)
C13—Sb—C7—C12	−5.8 (9)	Sb—C13—C14—C15	−179.0 (10)
O1^i^—Sb—C7—C12	81.6 (9)	C13—C14—C15—C16	−2(2)
O1—Sb—C7—C12	−93.3 (9)	C14—C15—C16—C15^i^	1.1 (10)
C7^i^—Sb—C7—C8	−7.2 (9)		

Symmetry codes: (i) −*x*+1, −*y*+1, *z*.

### Hydrogen-bond geometry (Å, °)

**Table:**

*D*—H···*A*	*D*—H	H···*A*	*D*···*A*	*D*—H···*A*
C10—H10···Br^ii^	0.93	2.90	3.69 (2)	144

Symmetry codes: (ii) −*x*+1/2, −*y*+1/2, *z*.
